# Inactivation of Myostatin Delays Senescence via TREX1-SASP in Bovine Skeletal Muscle Cells

**DOI:** 10.3390/ijms25105277

**Published:** 2024-05-12

**Authors:** Miaomiao Yang, Li Gao, Yajie Gao, Zhenting Hao, Xinyu Zhou, Guanghua Su, Chunling Bai, Zhuying Wei, Xuefei Liu, Lei Yang, Guangpeng Li

**Affiliations:** State Key Laboratory of Reproductive Regulation and Breeding of Grassland Livestock, College of Life Science, Inner Mongolia University, Hohhot 010021, China; 21708008@mail.imu.edu.cn (M.Y.); gaoli8905@163.com (L.G.); gaoyajie1997@163.com (Y.G.); 22208017@mail.imu.edu.cn (Z.H.); zhouxinyufjb@163.com (X.Z.); suguanghua0707@163.com (G.S.); chunling1980_0@163.com (C.B.); weizhuying2008@126.com (Z.W.); liuxuefei1006@126.com (X.L.)

**Keywords:** myostatin, skeletal muscle cell, cell senescence, TREX1, cGAS-STING, SASP

## Abstract

The myostatin (*MSTN*) gene also regulates the developmental balance of skeletal muscle after birth, and has long been linked to age-related muscle wasting. Many rodent studies have shown a correlation between *MSTN* and age-related diseases. It is unclear how *MSTN* and age-associated muscle loss in other animals are related. In this study, we utilized *MSTN* gene-edited bovine skeletal muscle cells to investigate the mechanisms relating to *MSTN* and muscle cell senescence. The expression of *MSTN* was higher in older individuals than in younger individuals. We obtained consecutively passaged senescent cells and performed senescence index assays and transcriptome sequencing. We found that senescence hallmarks and the senescence-associated secretory phenotype (SASP) were decreased in long-term-cultured myostatin inactivated (MT-KO) bovine skeletal muscle cells (bSMCs). Using cell signaling profiling, *MSTN* was shown to regulate the SASP, predominantly through the cycle GMP-AMP synthase-stimulator of antiviral genes (cGAS-STING) pathway. An in-depth investigation by chromatin immunoprecipitation (ChIP) analysis revealed that *MSTN* influenced three prime repair exonuclease 1 (*TREX1*) expression through the SMAD2/3 complex. The downregulation of *MSTN* contributed to the activation of the MSTN-SMAD2/3-TREX1 signaling axis, influencing the secretion of SASP, and consequently delaying the senescence of bSMCs. This study provided valuable new insight into the role of *MSTN* in cell senescence in large animals.

## 1. Introduction

Myostatin (*MSTN*) is also known as GDF-8 [1]. Natural mutations of *MSTN* are associated with an increase in muscle mass [2]. Currently, multiple species of *MSTN* gene inactivation mutant animals have been obtained through gene editing technology [3,4,5], and all the *MSTN* mutant animals show “double-muscled” phenotypes. Inactivation of the *MSTN* gene can not only improve individual growth levels and exercise abilities but also increase muscle production in livestock and poultry production, generating more economic benefits [6]. *MSTN* inhibits the development of skeletal muscle before birth and regulates muscle tissue homeostasis in adult animals. In addition, because of the similarity to human physiology, anatomy, genetics, and size, animals raised for food are also useful animal models for human diseases [7]. 

Skeletal muscle tissue is composed of muscle fibers, which are primarily divided into two types based on the proportion of myosin heavy chain (MHC) isoforms: Type I (slow oxidative fibers) and Type II (fast glycolytic fibers) [8]. The extracellular matrix (ECM) connects cylindrical fibers. Each fiber is enveloped by an ECM layer, which includes the perimysium and the basal lamina. Collagen fibers interweave with extensive vascular and nerve networks to form the perimysium, which is the outermost membrane. The reticular layer, composed of collagen fibers, and the basal layer form the basal lamina, which is located between the perimysium and the plasma membrane [9]. The plasma membrane contains many proteins, such as the dystrophin–glycoprotein complex that is involved in connecting fibers and the ECM [9]. Skeletal muscle’s ability to regenerate itself steadily decreases with age [10]. The aging process is linked to a reduction in the number and function of satellite cells, which severely impairs or even eliminates the remodeling ability of skeletal muscle [11]. Hyaluronan (HA) is ubiquitously expressed in the extracellular matrix (ECM) of mammals [12]. Researchers have also explored the potential of hyaluronans (HAs) in promoting cell growth and proliferation, as well as rescuing cells under oxidative stress, through three different experimental setups, the study indicates that different hyaluronan-based gels have a protective effect in the recovery of stressed cells and muscle atrophy [13].According to previous studies, inhibiting myostatin is a potential way to alleviate age-related muscle atrophy [14,15].

The secretion of many inflammation factors, which is also known as SASP, has been identified in recent research as a characteristic of senescent cells [16]. This can affect the function of neighboring cells and tissues, promoting tissue inflammation and deficiency, which could cause cellular senescence. Studies revealed that 1,25(OH)2D3 deficiency could cause cellular senescence and SASP in skeletal muscle cells to induce sarcopenia [17]. The innate DNA sensing pathway is essential to regulating SASP and senescence [18]. The SASP is triggered by the abnormal activation of the cytoplasmic DNA sensing mechanism, the cyclic GMP-AMP synthase-stimulator of interferon genes (cGAS-STING) [19]. TREX1 is a 3′ DNA exonuclease. Reports have shown that the loss of *TREX1* results in the inability of cells to undergo a normal cell cycle, resulting in defects in the G1/S phase transition in cells [20,21]. At the same time, *MSTN* negatively regulates the process of the G1/S phase, thereby inhibiting the cell cycle and maintaining the quiescent state of satellite cells [22]. This, in turn, affects the proliferation of skeletal muscle cells. *MSTN* can affect the expression of the cell cycle through the downstream transcription factor SMAD. The knockout of *MSTN* promotes cell proliferation, and the overexpression of *MSTN* inhibits cell proliferation and DNA synthesis [23]. In pre-senescent cells, the nucleus effectively clears cytoplasmic DNA fragments through the action of *TREX1* and *DNase2*. However, in senescent cells, the expression of DNase is decreased, resulting in the accumulation of nuclear DNA in the cytoplasm [19]. Following *TREX1* depletion, there is an increased content of ssDNA in the cytoplasm [24]. Subsequently, this triggers intracellular DNA sensing mechanisms, which, in turn, activate signaling pathways for pro-inflammatory factors [25,26].

Although the respective roles of *MSTN* and SASP in skeletal muscle aging have been reported, the mechanism by which the *MSTN* gene influences SASP and delays the aging of skeletal muscle cells is still unclear. In this study, we used skeletal muscle cells from consecutive passages of *MSTN* gene-edited bovine to explore the role of the *MSTN* gene in skeletal muscle aging. This exploration is of significant importance for understanding the generation of animal muscle mass and overall organism health, as well as for the treatment of aging-induced related diseases.

## 2. Results

### 2.1. MSTN Gene Inactivation Delayed the Senescence of Long-Term Cultured bSMCs

**Figure 1:**
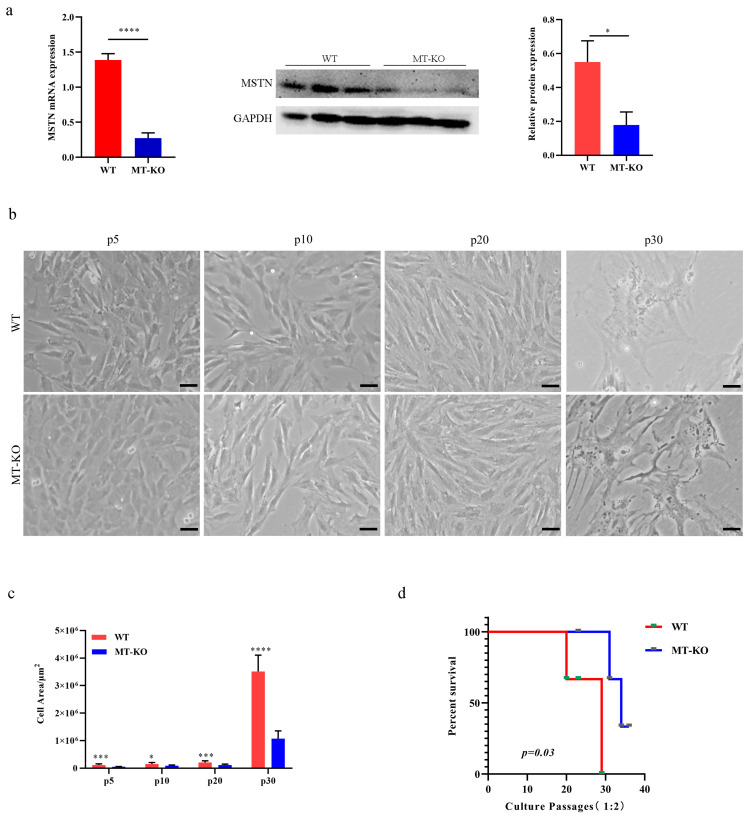
Characteristics of long-term cultured inactivation of the *MSTN* bSMCs. (**a**) Expression of MSTN mRNA and protein in bSMCs. (**b**) Muscle cells derived from bovine at different periods in the continuous subculture; scale bar, 1000 μm. (**c**) The difference in the area of passaged cells (μm^2^). (**d**) Survival curves of different generations in WT and MT-KO groups. The mean ± SD is used to present the data (n = 3). * *p* < 0.05; *** *p* < 0.001; **** *p* < 0.0001 (*t*-test).

To evaluate the effect of *MSTN* inactivation on the senescence of the long-term cultured bSMCs, we used WT and MT-KO bovine skeletal muscle cells (bSMCs) from our laboratory [27], where the expression of MSTN in MT-KO cells decreased more than in the WT cells (Figure 1a). Through a continuous passage, we obtained cells of different generations and established aging models of WT and MT-KO bSMCs. After many passages, WT and MT-KO cells tended to be flat, larger than the low passage cells, and they lacked plasticity (Figure 1b,c). The survival of cells through the number of passages was statistically plotted for both the WT and MT-KO groups, with the highest number of passages in the WT group being around 30 generations, while three cell lines in the MT-KO group were passed to less than 40 generations (Figure 1d).

### 2.2. Inactivation of the MSTN Alleviated Senescence Caused by the Long-Term Culture of bSMCs

**Figure 2:**
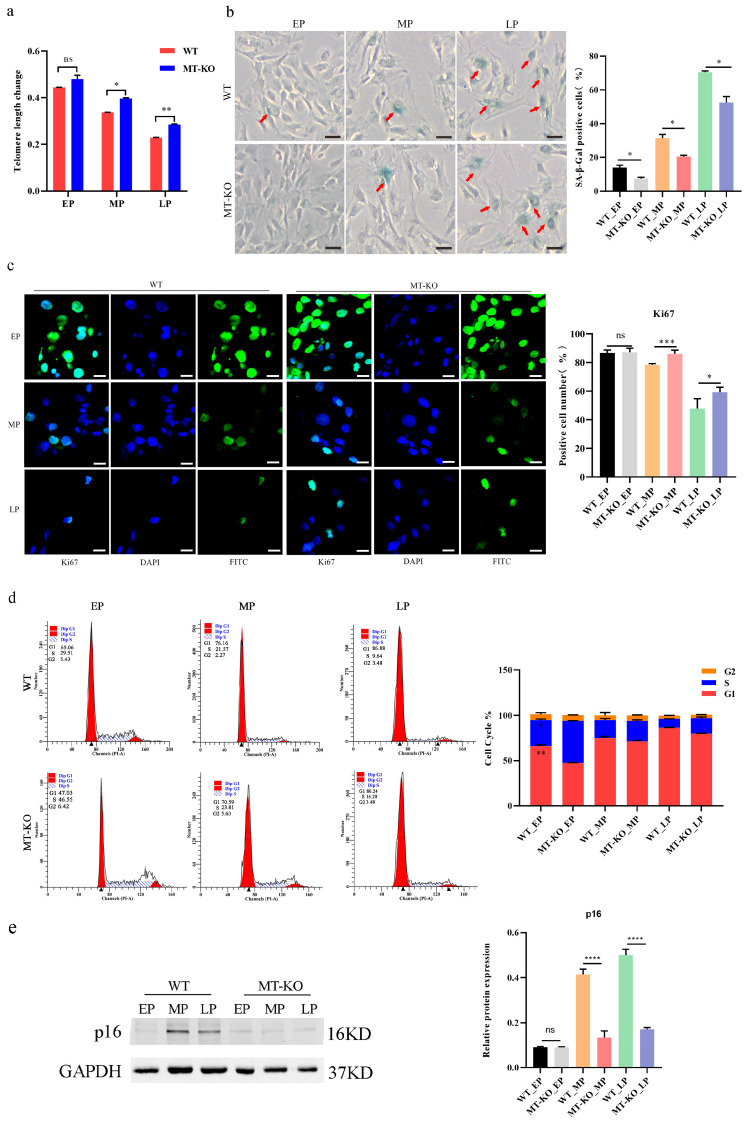
Hallmarks of the senescence phenotype in long-term-cultured bSMCs. (**a**) Changes in telomere length in skeletal muscle cells at different passage numbers. (**b**) SA-β-gal staining of WT and MT-KO cells and the percentages of SA-β-gal-positive cells of WT and MT-KO, the red arrows refer to the stained senescent cells. Scale bar, 1000 μm. (**c**) ki67 staining and ki67-positive cells percent. Scale bar, 50 μm. (**d**) Bar chart displaying the percentage of WT and MT-KO cells of the cell cycle. (**e**) Protein expression of p16 at different passage numbers. EP, MP, and LP stand for early passage, middle passage, and late passage, respectively. Early passage cells are around p10, middle passage cells are around p20, and late passage cells are around p30, same as below. The mean ± SD is used to present the data (n = 3). * *p* < 0.05; ** *p* < 0.01; *** *p* < 0.001; **** *p* < 0.0001; ns, *p* > 0.05 (*t*-test).

Although some advancement has been made in knowing the properties and mechanisms of senescent cells, researchers proposed the multi-marker method to assess the efficiency of cellular senescence; this method involves assessing senescence-associated beta-galactosidase (SA-β-gal) activity, co-staining markers typically related to cell proliferation, and p16, p21 expression, and elements anticipated to change in specific senescence scenarios [28]. We analyzed the senescence indicators in continuously passaged cells and compared the WT cells with the MT-KO cells. Telomere length decreased with the increase in passage number, and the telomere length in the WT cells was shorter than that of the MT-KO cells in LP cells (Figure 2a). We observed an increased percentage of cells positive for SA-β-gal staining (WT vs. MT-KO, EP 15.33% ± 2.52 vs. 8.33% ± 1.53; MP 32.67% ± 2.52 vs. 22% ± 2.65; LP 84% ± 3.61 vs. 74.33% ± 3.06) (Figure 2b), a decreased percentage of ki67-positive cells (WT vs. MT-KO, EP 89.69% ± 4.47 vs. 97.96% ± 0.37; MP 55.05% ± 0.44 vs. 84.96% ± 2.14; LP 18.86% ± 4.88 vs. 41.54% ± 2.22) (Figure 2c), a decrease in the G1/S-phase of the cell cycle (Figure 2d), and an upregulation of p16 protein expression (Figure 2e). These results showed that the downregulation of *MSTN* delayed cellular senescence in bovine skeletal muscle cells.

### 2.3. Inactivation of the MSTN Downregulated the SASP in Skeletal Muscle Senescence Cells

**Figure 3:**
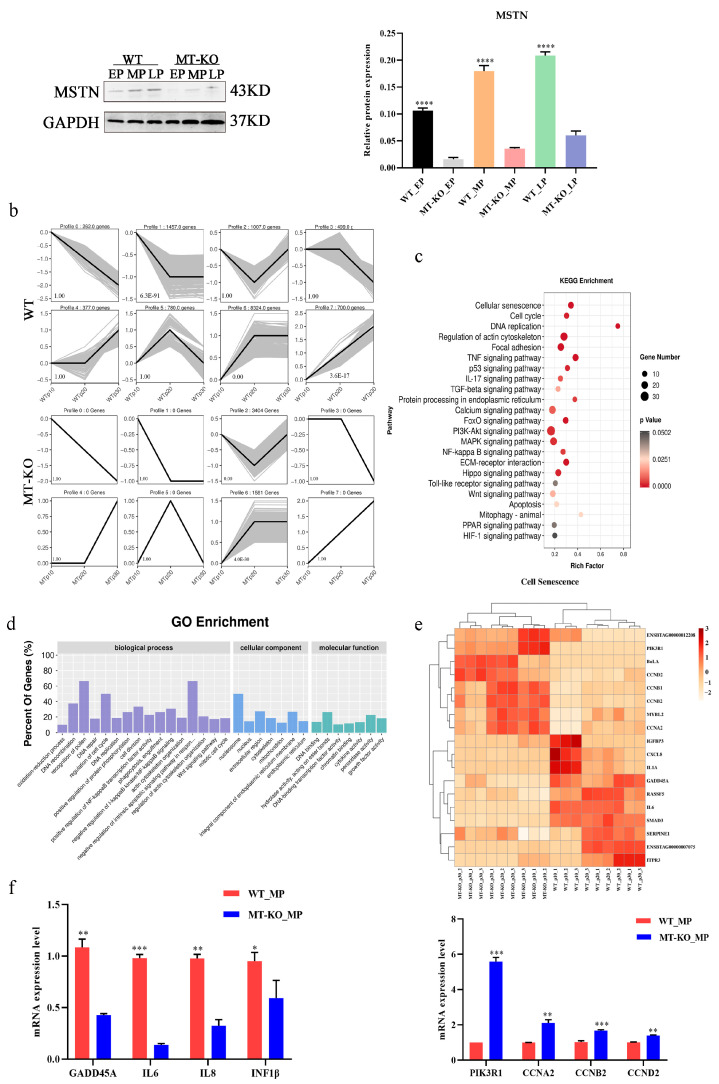
Inactivation of the *MSTN* downregulated the SASP in senescent bSMCs. (**a**) The expression of MSTN in cells with different passage numbers was examined using Western blotting. (**b**) STEM analysis: different colors represent significant differences, the number in the upper left corner indicates the profile number, the number in the upper right corner indicates the gene number, and the number in the bottom left corner indicates the significance of the differences. (**c**,**d**) Enriched KEGG, and GO pathways. (**e**) KEGG enrichment of cell senescence-associated genes. (**f**) The mRNA expression of increased genes and reduced genes in heatmap analysis. The mean ± SD is used to present the data (n = 3). * *p* < 0.05; ** *p* < 0.01; *** *p* < 0.001; **** *p* < 0.0001 (*t*-test).

Western blot analysis was used to detect the expression of the MSTN protein in the EP, MP, and LP of both WT and MT-KO cells. The results revealed an increase in MSTN expression with generation, yet significantly lower levels were observed in MT-KO cells compared to WT cells (Figure 3a). Then, we selected different stages of WT and MT-KO cells for transcriptome sequencing. To evaluate the dynamic changes in the trends of WT and MT-KO cells after continuous subculture, an analysis using genome-wide RNA sequencing (RNA-seq) was carried out. STEM analyses of WT (EP, MP, LP) and MT-KO (EP, MP, LP) cells were clustered into eight profiles for each sample. Profile 6 (8324), Profile 1 (1457), and Profile 7 (700) of the WT cells and Profile 2 (3404) and Profile 6 (1581) of the MT-KO cells were significantly different from the other profiles (Figure 3b). The genes with significant differences were analyzed using the GO and KEGG pathways (Figure 3c,d). The KEGG pathway analysis showed that 29, 726, and 894 pathways were mapped in EP, MP, and LP cells, of which 96, 917 pathways were significantly different, respectively (q value < 0.05), including cellular senescence, cell cycle, DNA replication, and the NF-κB signaling pathway (Figure 3c). A heatmap of the enriched genes related to cellular senescence was created and revealed that the GO terms “aging” and “SASP” were primarily linked to genes that were expressed differently in WT and MT-KO cells (Figure 3d,e). The mRNA expression of increased genes and reduced genes in the heatmap of SASP factors were analyzed (Figure 3f). These results indicate that the inactivation of the *MSTN* gene downregulated the SASP in skeletal muscle senescence cells. The basis of MSTN expression in different generations of cells was analyzed, and MP cells were selected for subsequent experiments.

### 2.4. Inactivation of the MSTN Downregulated the SASP through the Upregulation of TREX1

**Figure 4:**
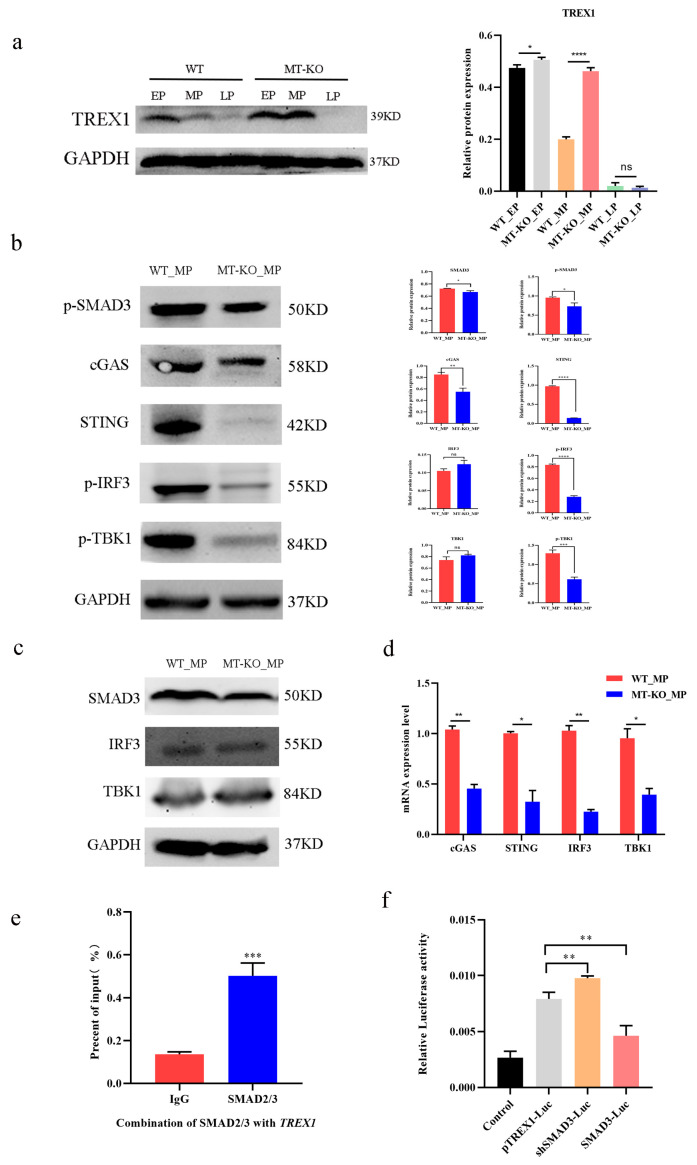
Inactivation of the *MSTN* downregulated the SASP through the upregulation of TREX1. (**a**) Protein expression of TREX1 in cells of WT and MT-KO with different passage numbers. (**b**–**d**) cGAS-STING downstream protein expression and mRNA expression of WT_MP and MT-KO_MP. (**e**) For ChIP evaluation, we utilized an amplified anti-SMAD2/3 monoclonal antibody targeting the *TREX1* binding region. (**f**) Dual luciferase assay showing SMAD3 negatively regulates *TREX1* expression. The mean ± SD is used to present the data (n = 3). * *p* < 0.05; ** *p* < 0.01; *** *p* < 0.001; **** *p* < 0.0001; ns, *p* > 0.05 (*t*-test).

According to reports, the innate DNA sensing system is essential to regulate the SASP and senescence [29]. Compared with the WT long-term-cultured bSMCs, TREX1 expression was increased in the MT-KO cells, and with the increase in generation, TREX1 expression was lower than in the early passages of WT and MT-KO (Figure 4a). In addition, the downstream factors of the cGAS-STING pathway were observed to decrease in the MT-KO cells (Figure 4b–d). Through the above, in bSMCs, MT-KO will affect the expression of TREX1, but how *MSTN* affects TREX1 needs to be explored. As SMAD2/3 acts as a downstream complex transcription factor of *MSTN*, we investigated its binding ability to the *TREX1* promoter region. Through prediction analysis using the JASPAR database, it was discovered that SMAD2/3 is linked to the TREX1 (NC_037349.1) promoter region between −1446 and −1437. The ChIP-qPCR findings indicated that there is binding of SMAD2/3 to the TREX1 promoter (Figure 4e). Using the luciferase reporter assay, we were able to detect transcriptional activity following SMAD3 overexpression to investigate the impact of SMAD2/3 on the transcriptional activity of *TREX1*. It was discovered that SMAD3 inhibits the TREX1 promoter’s transcriptional activity, and, after interfering with SMAD3, the activity of the *TREX1* promoter was significantly upregulated (Figure 4f). These results suggest that SMAD3 has a negative regulatory effect on the *TREX1* promoter.

### 2.5. Overexpression of TREX1 Can Rejuvenate Aged bSMCs

**Figure 5:**
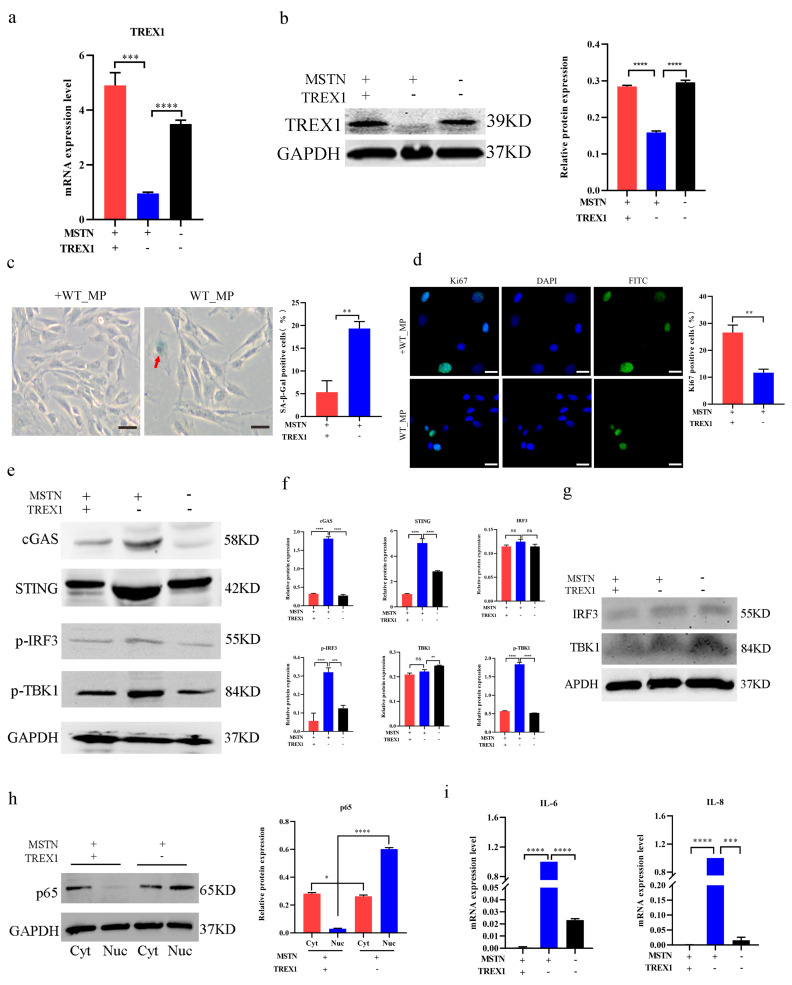
Overexpression of *TREX1* can rejuvenate aged bSMCs. (**a,b**) TREX1 mRNA and protein expression of overexpression TREX1 WT_MP group and WT_MP group. (**c**) SA-β-gal staining and positive cell percent of overexpression of *TREX1* WT_MP and WT_MP cells, the red arrows refer to the stained senescent cells. scale bar, 1000 μm. (**d**) ki67 staining and positive cell percent of overexpression of *TREX1* WT_MP and WT_MP cells; scale bar, 50 μm. (**e**–**g**) Protein expression of cGAS, STING, IRF3, p-IRF3, TBK1, and p-TBK1 in *TREX1* WT_MP and WT_MP cells. (**h**) Nuclear and cytoplasmic protein expression of p65 in *TREX1* WT_MP and WT_MP cells. (**i**) *IL-6* and *IL-8* mRNA expression in *TREX1* WT_MP and WT_MP cells. The mean ± SD is used to present the data (n = 3). * *p* < 0.05; ** *p* < 0.01; *** *p* < 0.001; **** *p* < 0.0001; ns, *p* > 0.05 (*t*-test).

To determine if *TREX1* regulated the rejuvenation of aged bSMCs, we transfected wild type bSMCs with an overexpression vector. As anticipated, the transgenic cells showed a notable increase in TREX1 expression (Figure 5a,b). The percent of SA-β-gal was decreased (5.33% ± 2.52 vs. 19.33% ± 1.53) (Figure 5c), and there was an increase in ki67-positive proliferating cells (26.6% ± 2.75 vs. 10.14% ± 1.2) (Figure 5d). For the protein expression of the downstream genes cGAS, STING, p-IRF3, p-TBK1, and *IL-6, IL-8* mRNA expression decreased significantly (Figure 5e,f,i), while the IRF3 and TBK1 total protein expression levels showed no obvious difference (Figure 5f,g), and the overexpression of *TREX1* resulted in lower p65 protein expression in the nucleus than in the cytoplasm (Figure 5h). As expected, the re-introduction of *TREX1* attenuated *cGAS, STING*, and the SASP response, and rescued the phenotype of accelerated bSMCs senescence.

### 2.6. Aging Prematurely Due to TREX1 Absence in bSMCs

**Figure 6:**
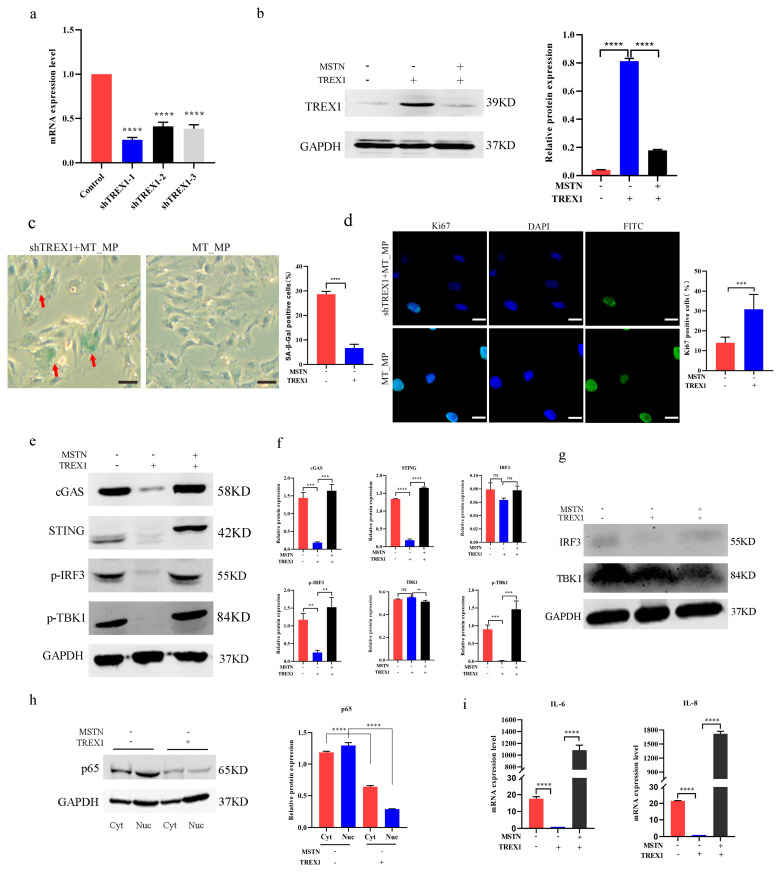
*TREX1* deficiency resulted in premature aging of bSMCs. (**a**) Transfected the TREX1 shRNA in MT-KO_MP bSMCs and evaluated the efficiency of interference. (**b**) TREX1 protein expression after deficiency of TREX1 in MT-KO_MP in bSMCs. (**c**) SA-β-gal staining and positive cells with percent of shTREX1_MP and MT-KO_MP cells, the red arrows refer to the stained senescent cells. scale bar, 1000 μm. (**d**) Ki67 staining and positive cell percent of shTREX1 and MT-KO cells; scale bar, 50 μm. (**e**–**g**) Protein expression of cGAS, STING, IRF3, p-IRF3, TBK1, and p-TBK1, of shTREX1_MP and MT-KO_MP cells. (**h**) Nuclear and cytoplasmic protein expression of p65 of shTREX1_MP and MT-KO_MP cells. (**i**) mRNA expression of *IL-6* and *IL-8*. The mean ± SD is used to present the data (n = 3). ** *p* < 0.01; *** *p* < 0.001; **** *p* < 0.0001; ns, *p* > 0.05 (*t*-test).

To investigate the effect of *TREX1* deficiency in MT-KO_MP bSMCs, we transfected the TREX1 shRNA in MT-KO_MP bSMCs and evaluated the efficiency of interference. shTREX-1, with the highest interference efficiency, was selected for subsequent experiments (Figure 6a). The loss of TREX1 protein expression in TREX1-deficient MT-KO_MP bSMCs was verified by Western blotting (Figure 6b). There were higher proportions of SA-β-gal in the TREX1-deficient cells than in the MT-KO_MP cells (28.67% ± 1.15 vs. 6.67% ± 1.53) (Figure 6c), and a lower proportion of ki67-positive cells (13.95% ± 2.85 vs. 30.83% ± 7.47) (Figure 6d). The protein expression of downstream genes, including cGAS, STING, p-TBK1, and p-IRF3, exhibited a significant increase (Figure 6e,f), and the IRF3, and TBK1 total protein expression levels showed no obvious difference (Figure 6f,g). In shTREX1 MT-KO_MP bSMCs, p65 protein expression was higher in the nucleus than in the cytoplasm (Figure 6h). The mRNA expression of the SASP-related genes *IL-6* and *IL-8* was higher in the TREX1-deficient cells than in the MT-KO_MP cells (Figure 6i).

## 3. Discussion

The skeletal muscle, as the largest organ in the human body, is essential for the survival of the organism and performs a variety of functions [30]. Although it has great regenerating capacity, after damage, this capacity decreases with age. With age increase, interventions such as exercise will not be able to help one avoid muscle atrophy as well as decreased muscle mass due to other diseases [31]. Baumann et al. discovered that the myostatin protein content increased with age from 1.5 to 27 months in the rat’s gastrocnemius muscle [32]. The levels of myostatin and SA-β-gal in mouse skeletal muscle increased with age [33]. The knockdown of myostatin reveals significant muscle hypertrophy. Throughout the process of development, satellite cell-derived myoblasts multiply and differentiate continually before fusing with pre-existing muscle fibers to enlarge them [34]. Compared to wild-type animals, adult Mstn^−/−^ mice exhibit a notable rise in both the number of satellite cells per muscle fiber and the percentage of activated satellite cells. Introducing myostatin shRNAs via injection in rats led to a more than twofold augmentation in satellite cell numbers [22].

For the study of cellular senescence, the most commonly used method is to continuously pass the cells to obtain a replicative cellular senescence model [35]. This experiment was based on this continuous passaging culture until the cells were difficult to collect. Compared to low-passage cells, high-passage cells from both the WT and MT-KO groups exhibited significant morphological changes. Studies have reported significant changes in cell phenotype, differentiation potential, and gene expression after passages [36,37]. Therefore, we examined telomere length, SA-β-gal activity, ki67-positive cells, cell cycle dynamics, and the expression of p16 with increasing passage numbers. Both the WT and MT-KO groups exhibited noticeable changes in these aging-related indicators as the passage number increased. Through the detection of these aging indicators, we confirmed the continuously passaged senescent cells were suitable for subsequent experiments. Then, transcriptome sequencing analysis was conducted on the obtained continuously passaged senescent cells, and the result highlighted genes related to cellular senescence. We analyzed these genes and found that most of them were associated with the SASP. It is increasingly evident that the release of inflammatory mediators is one aspect of aging [38]. Senescent cells demonstrate cell cycle arrest [39], tissue repair ability loss [40], and proinflammatory cytokine release [41]. Several mechanisms have been demonstrated to be involved in regulating the activity of SASP factors, including transcription-related variables, positive regulators of SASP factor expression Nuclear Factor κB (NF-κB) [42], and the cGAS-STING signaling pathway. DNA damage leads to the accumulation of damaged DNA in the cytoplasm of cells containing cGAS, which is regarded as a cytoplasmic DNA sensor [43]. It can identify and detect damage to DNA, and when cytoplasmic DNA (mtDNA, cDNA, and pieces of cytoplasmic chromatin) accumulates in senescent cells, it produces cGAMP, which sets off the SASP. In inflammatory conditions, aberrant DNA metabolism and the proinflammatory impact of cfDNA buildup may be exacerbated by decreased TREX1 expression, which could ultimately cause normal cells to progressively acquire the SASP [44].

**Figure 7:**
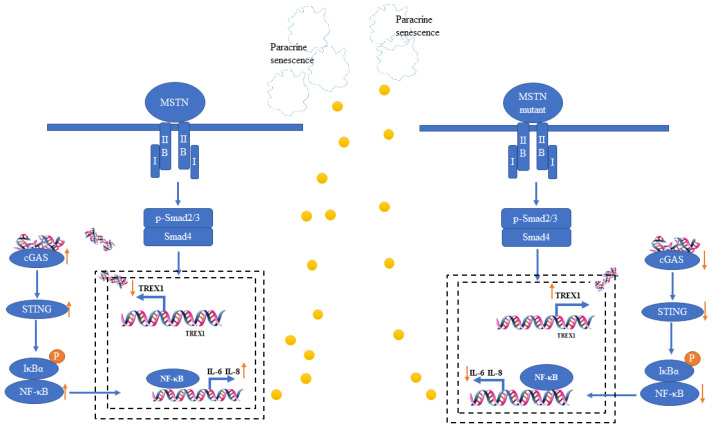
Schematic of the mechanism of MT-KO in bSMCs senescence. In the *MSTN* inactivation model, *MSTN* formed a complex and ActRIIB then combined with the type I receptor. The activated MT-KO signal then activated phosphorylated SMAD2/3, where up on SMAD2/3 it was decreased and combined with SMAD4. By combining with the *TREX1* promoter inside the nucleus, this compound enhanced TREX1’s activity. The increase in *TREX1* reduced the damaged DNA in the cytoplasm, decreased cGAS-STING signaling, decreased secretion of the SASP factors, and delayed senescence.

Combining transcriptome data, we conducted an expression-level analysis of *TREX1* at different passages and found that with increasing passages, both TREX1 mRNA and protein expression decreased. In the WT group, the decrease occurred after EP, while in the experimental group, it occurred after MP. TREX1-deficient mice induce autoimmune disorders by activating downstream signaling pathways including the cGAS [45]. We simultaneously detected the mRNA and protein expression levels of cGAS and STING downstream of TREX1. As compared to the WT group, the MT-KO group’s expression levels were lower, according to the data. This result is in line with earlier studies. However, the regulatory effect of *MSTN* on *TREX1* and cGAS-STING signaling pathways remains unclear. Then, we explored the potential association between *MSTN* absence and *TREX1*. The results indicate that *MSTN* regulates *TREX1* through SMAD2/3. SASP-phenotyping senescent cells that express GATA4 and NF-κB factors produce IL-6 and IL-8, proteases, and matrix metalloproteinases [46]. p65 is an important member of the NF-κB transcription factor family, playing crucial roles in both the cytoplasm and the nucleus, undergoing dynamic translocation under different environmental and conditional cues [47]. In its inactive state, p65 exists in the cytoplasm, and when it becomes active, it moves to the nucleus and takes part in biological processes such as the immune response, inflammation, and cell proliferation [48]. To further confirm this, *TREX1* was overexpressed in the WT group, and *TREX1* was interfered with in the MT-KO group. It was found that after *MSTN* inactivation, by modulating the activity of SMAD2/3 and *TREX1*, the downstream cGAS-STING signaling pathway was affected, influencing the secretion of SASP, and consequently delaying the senescence of bMSCs cells (Figure 7). Conversely, *MSTN* skeletal muscle cells are more susceptible to replicative senescence effects.

In this study, we only discussed nuclear DNA. When DNA is in the cytoplasm, the DNA sensing mechanism is activated, thereby activating the pro-inflammatory cytokine pathway [20,21,49]. Mitochondrial DNA exists in the mitochondrial matrix and is a circular molecule of double-stranded dsDNA, adjacent to the electron transport chain, which is the main source of reactive oxygen species. Therefore, it is particularly susceptible to oxidation, leading to mtDNA mutations, which may lead to disease and aging [50]. The application of mtDNA and cGAS-STING signals in skeletal muscle aging deserves further exploration.

## 4. Materials and Methods

### 4.1. Cell Culture and Isolation

Three 2-year-old *MSTN*^+/−^ (MT-KO) and three wild-type (WT) heifer bovine skeletal muscle cells from our laboratory were chosen [27]. Wild-type and *MSTN*^+/−^ bovine muscle tissues were obtained after they were slaughtered, and then the tissues were rinsed in PBS with 2% penicillin–streptomycin (Gibco, Grand Island, NY, USA) three times, treated with 75% ethanol, and followed by treatment with PBS three times. The tissues were treated with 1 mg/mL collagenase IV (Thermo Fisher Scientific, Waltham, MA, USA) for 2–3 h to digest into cells and were cultured using DMEM medium (Thermo Fisher Scientific, Waltham, MA, USA) containing 20% fetal bovine serum (FBS, Thermo Fisher Scientific, Waltham, MA, USA) and 10% horse serum (HS, Thermo Fisher Scientific, Waltham, MA, USA) at 38.5℃ with 5% CO_2_. The obtained cells were cultured and passage culture was performed when the cell confluence reached 90%until the cells were difficult to collect and the passage was finished [27]. Due to the difficulty in sampling cells after more than 30 generations, cells from the 30-generation period were chosen as the late passage (LP). Cells from the p10-generation period were taken as early passage (EP) samples, and cells from the p20-generation period were taken as middle-passage (MP) samples for experimentation.

### 4.2. Telomere Length Assay

DNA of the different generation cells was extracted. PCR was performed to amplify specific segments of both telomere DNA and the single-copy gene. The threshold cycle (CT) values for telomere DNA and the single-copy gene were detected. ΔCT was calculated by subtracting the CT value of the single-copy gene from that of telomere DNA. The formula T/S = 2^−ΔCT^ was used to calculate the T/S ratio. 

### 4.3. SA-β-Gal Staining

Cells of different generations were first fixed with fixative (0.2% glutaraldehyde and 2% formaldehyde mixture) for 5 min at 25 °C, and then stained using SA-β-gal staining solution (Beyotime, shanghai, China, C0602) overnight at 25 °C. Finally, the percentage of cells positive for SA-β-gal was counted.

### 4.4. Immunofluorescent Staining

Cells from various generations were fixed using 4% paraformaldehyde for 30 min at 25 °C. Subsequently, they were rinsed 3 times with PBS and then incubated overnight at 4 °C with anti-Ki67 antibodies (Abcam, Cambridge, MA, USA, ab15580, diluted 1:500). After another three washes with PBS solution, the cells were incubated with secondary antibodies for 15 min at 25 °C. Finally, nuclear staining was performed using DAPI. Images were captured using a confocal microscope (A1R, Nikon, Tokyo, Japan).

### 4.5. The use of Fluorescence-Activated Cell Sorting for Cell Cycle Assay (FACS)

Cells of different generations were collected into a centrifuge tube and resuspended using ice-cold PBS. They were fixed for 30 min at 4 °C using cold 70% ethanol, and then washed with PBS followed by propidium iodide (PI) staining, following the instructions in the assay kit (Beyotime, shanghai, China, C1052). Then, they were incubated at 37 °C for 30 min in the dark. The treated cells were detected by flow cytometry (ModFIT LT v3.1) for cell cycle analysis within 24 h.

### 4.6. Real-Time PCR

Using the RNAiso Plus kit, the total RNA of cells of different generations was extracted (Takara, Tokyo, Japan). Next, the RNA was reverse transcribed into cDNA. The expression of genes was detected by real-time qPCR with SYBR Green fluorescence dye (Takara, Tokyo, Japan) using LC480 (Rocho, Basel, Switzerland). Supplementary Table S1 contains the PCR primers that Sangon Biotech generated. The PCR amplification protocol was as below: 95 °C for 30 s, 95 °C for 5 s, and 60 °C for 34 s for 40 cycles. Using GAPDH as the reference gene, the relative expression of genes was calculated using the 2^−ΔΔCt^ method.

### 4.7. Western Blot

Western Blot experiments were performed using SDS-polyacrylamide gel electrophoresis. Then, RIPA lysis buffer was used for extraction, the protein of cells was then separated using 10% SDS-polyacrylamide gels, and then the protein was transferred to the NC membranes. Then, it was blocked using 0.5% skim milk. It was incubated overnight at 4 °C with the corresponding primary antibody. Antibodies were as below: anti-GDF8 (Abcam, Cambridge, MA, USA, ab201954, 1:1000), anti-TREX1 (Proteintech, Wuhan, China, 24876-1-AP, 1:500), anti-cGAS (Proteintech, Wuhan, China, 26416-1-AP, 1:500), anti-STING (Proteintech, Wuhan, China, 19851-1-AP, 1:500), TBK1 (Proteintech, Wuhan, China, 28397-1-AP, 1:500), IRF3 (Proteintech, Wuhan, China, 11312-1-AP, 1:500), p16 (Proteintech, Wuhan, China, 10883-1-AP, 1:500), p21 (Proteintech, Wuhan, China, 10355-1-AP, 1:500), IL-6 (Proteintech, Wuhan, China, 21865-1-AP, 1:500), IL-8 (Proteintech, Wuhan, China, 17038-1-AP, 1:500), p-SMAD3 (Abcam PIC, Cambridge, MA, USA, ab52903, 1:1000), p-TBK1 (Cell Signaling Technology, Danvers, MA, USA, 5483T, 1:1000), p-IRF3 (Cell Signaling Technology, Danvers, MA, USA, 37829T, 1:1000), p65 (Santa Cruz, Santa Cruz, CA, USA, SC8008, 1:200), and GAPDH (Proteintech, China, Wuhan, 60004-1-Ig, 1:1000). The corresponding secondary antibody was subsequently incubated for 1 h at 37 °C. Finally, ECL Plus (Thermo Fisher Scientific, Waltham, MA, USA, 32209) was used for color development. Quantitative analysis was performed using ImageJ (1.8.0).

### 4.8. RNA-Seq Analysis

The total RNA of cells of different generations was isolated and purified using the TRIzol kit (Thermo Fisher Scientific, Waltham, MA, USA), and RNA integrity was assessed and confirmed by agarose gel electrophoresis. Then, poly (A) RNA purification, RNA fragmentation, reverse transcription, second-strand DNA synthesis, A-tailing and adapter ligation, size choosing, and PCR amplification were carried out. The average insert size was 300 ± 50 bp for the final cDNA library. Subsequently, the vendor’s recommended methodology was followed to perform 2 × 150 bp paired-end sequencing (PE150) on an Illumina NovaseqTM 6000 (LC-Bio Technology CO., Ltd., Hangzhou, China). A HISAT2 software (https://daehwankimlab.github.io/hisat2/, version:hisat2-2.0.4) reads genome and StringTie (http://ccb.jhu.edu/software/stringtie/, version:stringtie-1.3.4d.Linux_x86_64) were used to build each sample’s mapped readings. PCA and STEM data analysis were carried out by the omicshare software (https://www.omicshare.com/, accessed on 8 March 2024). KEGG, GO, and heatmap data analysis were carried out by the omicstudio software (https://www.omicstudio.cn/index, accessed on 10 March 2024). 

### 4.9. Chromatin Immunoprecipitation Assay

ChIP assays were carried out using kits (Thermo Fisher Scientific, USA). Briefly, cells were fixed using formaldehyde and quenched using glycine. MNase was used to lyse and digest DNA. Cells were subsequently sonicated to break the nuclear envelope. The resulting lysates were subsequently incubated overnight at 4 °C with SMAD2/3 antibodies (Abcam, USA, ab202445) and protein G beads. After that, DNA was restored with the use of a DNA purification kit. Quantitative PCR was used to determine the purity of the DNA.

### 4.10. Luciferase Reporter

The amplified TREX1 gene promoter region from bovine genomic DNA was integrated with the pGL3-Basic vector (Promega, Madison, WI, USA) and transfected to the seeded HEK293T. Then, 48 h later, luciferase activity was assessed by a dual-luciferase reporter assay system (Promega, USA).

### 4.11. Transfection with TREX1 shRNA and Overexpression Vector

TREX1 shRNA (pSGU6/GFP/Neo-TREX1-cattle-896, pSGU6/GFP/Neo-TREX1-cattle-1288, pSGU6/GFP/Neo-TREX1-cattle-683) and control shRNA (pSGU6/GFP/Neo-shNC) were purchased from Sangon Biotech and the sequence is listed in Table S1. The cDNA of bovine, as a template to clone to obtain the TREX1 gene, and the overexpression vector pCAG-FLAG-TREX1 were constructed by insertion into the pCAG-IRES-eGFP vector by EcoRI and BamHI after correct sequencing. Lipofectamine 2000 Reagent was used to transfect the shRNA and overexpression vectors (Thermo Fisher Scientific, Waltham, MA, USA).

### 4.12. Statistics Analysis

The mean ± SD of three different experiments was used to express all the data. The error bars in the graphs represent one standard deviation, whereas the bars in the graphs represent the means. For statistical analysis, Welch’s *t*-test was employed to compare data from two groups with various standard deviations. A recurring one-way ANOVA was utilized when comparing two or more groups. ∗ *p* <0.05 indicated statistically significant results. The phototypesetting was carried out in Adobe Photoshop CS3, and the histograms were created in Prism 8.0 (GraphPad, La Jolla, CA, USA).

## 5. Conclusions

In this study, through the continuous passaging of bovine skeletal muscle cells, a replicative senescence model of bovine skeletal muscle cells was established, and an analysis of the transcriptome data revealed that they were mainly genes associated with the SASP. *MSTN* regulates the SASP through the MSTN-SMAD2/3-TREX1 signaling axis. The overexpression of *TREX1* in the WT group delayed the aging process in accelerated aging skeletal muscle cells. It interfered with the *TREX1* MT-KO group, accelerating the aging of skeletal muscle cells. This study expands the current knowledge on *MSTN* and skeletal muscle cell senescence, demonstrating that the inactivation of *MSTN* is involved in delaying the aging of bovine skeletal muscle cells.

## Data Availability

The BIG Data Center’s Genome Sequencing Archive holds the raw sequencing data presented in this paper. Publicly available data can be found at the Beijing Institute of Genomics (BIG), Chinese Academy of Sciences, accession number CRA004152, https://bigd.big.ac.cn/gsa, accessed on 30 April 2021.

## Supplementary Materials

The following supporting information can be downloaded at https://www.mdpi.com/article/10.3390/ijms25105277/s1.

## References

[1] McPherron A.C., Lawler A.M., Lee S.J. (1997). Regulation of skeletal muscle mass in mice by a new TGF-beta superfamily member. Nature.

[2] Williams M.S. (2004). Myostatin mutation associated with gross muscle hypertrophy in a child. N. Engl. J. Med..

[3] Paek H.J., Li Z.Y., Quan B.H., Yin X.J. (2023). Application of PCR-RFLP for quick identification of MSTN mutants in MSTN mutant pig breeding. Anim. Biotechnol..

[4] Gu M., Wang S., Di A., Wu D., Hai C., Liu X., Bai C., Su G., Yang L., Li G. (2023). Combined Transcriptome and Metabolome Analysis of Smooth Muscle of Myostatin Knockout Cattle. Int. J. Mol. Sci..

[5] Song S.Z., He Z.Y., Cheng Y., Yu B.L., Zhang T., Li D. (2022). MSTN modification in goat mediated by TALENs and performance analysis. Yi Chuan.

[6] Hai C., Bai C., Yang L., Wei Z., Wang H., Ma H., Ma H., Zhao Y., Su G., Li G. (2023). Effects of Different Generations and Sex on Physiological, Biochemical, and Growth Parameters of Crossbred Beef Cattle by Myostatin Gene-Edited Luxi Bulls and Simmental Cows. Animal.

[7] Petersen B. (2023). Editorial: Genetic engineering in farm animals. Front. Genet..

[8] Schiaffino S., Reggiani C. (2011). Fiber types in mammalian skeletal muscles. Physiol. Rev..

[9] Sanes J.R. (2003). The basement membrane/basal lamina of skeletal muscle. J. Biol. Chem..

[10] Piegari E., Angelis A.D., Cappetta D., Russo R., Esposito G., Costantino S., Graiani G., Frati C., Prezioso L., Berrino L. (2013). Doxorubicin induces senescence and impairs function of human cardiac progenitor cells. Basic. Res. Cardiol..

[11] Barani A.E., Durieux A.C., Sabido O., Freyssenet D. (2003). Age-related changes in the mitotic and metabolic characteristics of muscle-derived cells. J. Appl. Physiol..

[12] Fakhari A., Berkland C. (2013). Applications and emerging trends of hyaluronic acid in tissue engineering, as a dermal filler and in osteoarthritis treatment. Acta Biomater..

[13] Stellavato A., Abate L., Vassallo V., Donniacuo M., Rinaldi B., Schiraldi C. (2020). An in vitro study to assess the effect of hyaluronan-based gels on muscle-derived cells: Highlighting a new perspective in regenerative medicine. PLoS ONE.

[14] Siriett V., Platt L., Salerno M.S., Ling N., Kambadur R., Sharma M. (2006). Prolonged absence of myostatin reduces sarcopenia. J. Cell Physiol..

[15] Mendias C.L., Bakhurin K.I., Gumucio J.P., Shallal-Ayzin M.V., Davis C.S., Faulkner J.A. (2015). Haploinsufficiency of myostatin protects against aging-related declines in muscle function and enhances the longevity of mice. Aging Cell.

[16] Ruiz de Galarreta M., Lujambio A. (2017). DNA sensing in senescence. Nat. Cell Biol..

[17] Wang Q., Zhao J., Chen H., Zhou J., Chen A., Zhang J., Wang Y., Mao Z., Wang J., Qiu X. (2023). Bmi-1 Overexpression Improves Sarcopenia Induced by 1,25(OH)_2_D_3_ Deficiency and Downregulates GATA4-Dependent Rela Transcription. J. Bone Min. Res..

[18] Glück S., Ablasser A. (2019). Innate immunosensing of DNA in cellular senescence. Curr. Opin. Immunol..

[19] Takahashi A., Loo T.M., Okada R., Kamachi F., Watanabe Y., Wakita M., Watanabe S., Kawamoto S., Miyata K., BaRber G.N. (2018). Downregulation of cytoplasmic DNases is implicated in cytoplasmic DNA accumulation and SASP in senescent cells. Nat. Commun..

[20] Yang Y.G., Lindahl T., Barnes D.E. (2007). Trex1 exonuclease degrades ssDNA to prevent chronic checkpoint activation and autoimmune disease. Cell.

[21] Atianand M.K., Fitzgerald K.A. (2013). Molecular basis of DNA recognition in the immune system. J. Immunol..

[22] McCroskery S., Thomas M., Maxwell L., Sharma M., Kambadur R. (2003). Myostatin negatively regulates satellite cell activation and self-renewal. J. Cell Biol..

[23] Taylor W.E., Bhasin S., Artaza J., Byhower F., Azam M., Willard D.H., Kull F.C., Gonzalez-Cadavid N. (2001). Myostatin inhibits cell proliferation and protein synthesis in C2C12 muscle cells. Am. J. Physiol. Endocrinol. Metab..

[24] Erdal E., Haider S., Rehwinkel J., Harris A.L., McHugh P.J. (2017). A prosurvival DNA damage-induced cytoplasmic interferon response is mediated by end resection factors and is limited by Trex1. Genes. Dev..

[25] Harding S.M., Benci J.L., Irianto J., Discher D.E., Minn A.J., Greenberg R.A. (2017). Mitotic progression following DNA damage enables pattern recognition within micronuclei. Nature.

[26] Mackenzie K.J., Carroll P., Martin C.A., Murina O., Fluteau A., Simpson D.J., Olova N., Sutcliffe H., Rainger J.K., Leitch A. (2017). cGAS surveillance of micronuclei links genome instability to innate immunity. Nature.

[27] Gao L., Yang M., Wei Z., Gu M., Li G. (2020). MSTN Mutant Promotes Myogenic Differentiation by Increasing Demethylase TET1 Expression via the SMAD2/SMAD3 Pathway. Int. J. Biol. Sci..

[28] Gorgoulis V., Adams P.D., Alimonti A., Bennett D.C., Bischof O., Bishop C., Campisi J., Collado M., Evangelou K., Ferbeyre G. (2019). Cellular Senescence: Defining a Path Forward. Cell.

[29] Glück S., Guey B., Gulen M.F., Wolter K., Kang T.W., Schmacke N.A., Bridgeman A., Rehwinkel J., Zender L., Ablasser A. (2017). Innate immune sensing of cytosolic chromatin fragments through cGAS promotes senescence. Nat. Cell Biol..

[30] Park S.Y., Kim H.Y., Lee J.H., Yoon K.H., Chang M.S., Park S.K. (2010). The age-dependent induction of apoptosis-inducing factor (AIF) in the human semitendinosus skeletal muscle. Cell. Mol. Biol. Lett..

[31] Etienne J., Liu C., Skinner C.M., Conboy M.J., Conboy I.M. (2020). Skeletal muscle as an experimental model of choice to study tissue aging and rejuvenation. Skelet. Muscle.

[32] Baumann A.P., Ibebunjo C., Grasser W.A., Paralkar V.M. (2003). Myostatin expression in age and denervation-induced skeletal muscle atrophy. J. Musculoskelet. Neuronal Interact..

[33] Gutierrez-Salmean G., Ciaraldi T.P., Nogueira L., Barboza J., Taub P.R., Hogan M.C., Henry R.R., Meaney E., Villarreal F., Ceballos G. (2014). Effects of (-)-epicatechin on molecular modulators of skeletal muscle growth and differentiation. J. Nutr. Biochem..

[34] Bischoff R., Heintz C. (1994). Enhancement of skeletal muscle regeneration. Dev. Dyn..

[35] Hayflick L. (1965). The limited in vitro lifetime of human diploid cell strains. Exp Cell Res..

[36] Wang S., Hu B., Ding Z., Dang Y., Wu J., Di L., Liu X., Xiao B., Zhang W., Ren R. (2018). ATF6 safeguards organelle homeostasis and cellular aging in human mesenchymal stem cells. Cell Discov..

[37] Wagner W., Horn P., Castoldi M., Diehlmann A., Bork S., Saffrich R., Benes V., Blake J., Pfister S., Eckstein V. (2008). Replicative senescence of mesenchymal stem cells: A continuous and organized process. PLoS ONE.

[38] Lasry A., Ben-Neriah Y. (2015). Senescence-associated inflammatory responses: Aging and cancer perspectives. Trends Immunol..

[39] Childs B.G., Gluscevic M., Baker D.J., Laberge R.M., Marquess D., Dananberg J., van Deursen J.M. (2017). Senescent cells: An emerging target for diseases of ageing. Nat. Rev. Drug Discov..

[40] He S., Sharpless N.E. (2017). Senescence in Health and Disease. Cell.

[41] Salama R., Sadaie M., Hoare M., Narita M. (2014). Cellular senescence and its effector programs. Genes. Dev..

[42] Kuilman T., Michaloglou C., Vredeveld L.C., Douma S., van Doorn R., Desmet C.J., Aarden L.A., Mooi W.J., Peeper D.S. (2008). Oncogene-induced senescence relayed by an interleukin-dependent inflammatory network. Cell.

[43] Zhixun Dou K.G.M.G.V.J.Z. (2017). Cytoplasmic chromatin triggers inflammation in senescence and cancer. Nature.

[44] Luo W.D., Wang Y.P., Lv J., Liu Y., Qu Y.Q., Xu X.F., Yang L.J., Lin Z.C., Wang L.N., Chen R.H. (2023). Age-related self-DNA accumulation may accelerate arthritis in rats and in human rheumatoid arthritis. Nat. Commun..

[45] Lan Y., LondoO D., Bouley R., Rooney M., Hacohen N. (2014). Dnase2a Deficiency Uncovers Lysosomal Clearance of Damaged Nuclear DNA via Autophagy. Cell Rep..

[46] Loeser R.F., Collins J.A., Diekman B.O. (2016). Ageing and the pathogenesis of osteoarthritis. Nat. Rev. Rheumatol..

[47] Park J., Jang K.M., Park K.K. (2022). Effects of Apamin on MPP(+)-Induced Calcium Overload and Neurotoxicity by Targeting CaMKII/ERK/p65/STAT3 Signaling Pathways in Dopaminergic Neuronal Cells. Int. J. Mol. Sci..

[48] Assis S.I.S., Amendola L.S., Okamoto M.M., Ferreira G.D.S., Iborra R.T., Santos D.R., Santana M.F.M., Santana K.G., Correa-Giannella M.L., Barbeiro D.F. (2024). The Prolonged Activation of the p65 Subunit of the NF-Kappa-B Nuclear Factor Sustains the Persistent Effect of Advanced Glycation End Products on Inflammatory Sensitization in Macrophages. Int. J. Mol. Sci..

[49] Muñoz-Espín D., Serrano M. (2014). Cellular senescence: From physiology to pathology. Nat. Rev. Mol. Cell Biol..

[50] Riley J., Tait S. (2020). Mitochondrial DNA in inflammation and immunity. EMBO Rep..

